# Author reply to “The importance of patients’ experience in assessing the quality of primary care”

**DOI:** 10.1002/jgf2.351

**Published:** 2020-06-28

**Authors:** Makiko Ozaki, Shinji Matsumura

**Affiliations:** ^1^ Murasakino Kyouritsu Clinic Kyoto Japan; ^2^ Division of Clinical Epidemiology National Hospital Organization Tokyo Medical Center Tokyo Japan; ^3^ Matsumura Clinic Tokyo Japan

To the Editor,

We thank the authors of the Letter to the Editor^1^ for their interest in our article, “Quality of primary care provided in community clinics in Japan”^2^ We completely agree with them that patient‐centeredness measured by patients’ experience is one of the important elements of quality of primary care, along with equitable, timely, safe, effective, and efficient care^3^ We also agree with the authors’ suggestion that Japanese healthcare policy should implement patients’ experience as Brazilian Federal Government do.

The reasons why we did not use the Japanese version of Patient Care Assessment Tool (PCAT) to measure quality of primary care in our study were following two.

First, in this study, we emphasized to measure technical care focused on process qualities in Donabedian's conceptual model^4^ Technical quality of care describes the extent to which the use of healthcare services meets predefined standards of acceptable or adequate care relative to need, that is, patient receipt of the recommended care. For this purpose, we used quality indicators as a measurement in our study. Quality indicators (QIs) have been used to measure quality of technical care and have been used to evaluate and improve the quality of healthcare services for nearly two decades worldwide^5^, ^6^. Those 18 QIs, out of 39 QIs of our QI set^7^ we used in this study, were mostly process‐oriented and were measurable using data resources other than patient survey. Although QIs are explicitly defined, and measurable items include structures, processes, or outcomes of care^8^, PCAT is to be a measurement mainly for patients’ experience, that is, outcomes of qualities of care.

Second, when we started our study, the Japanese version of PCAT had not been published unfortunately, therefore we were unable to use it at that time. Since now it is available for use, it may be useful to evaluate outcome of quality of primary care by using this validated instrument for international comparisons.

Evaluating process qualities by using QIs is time‐consuming, especially when we conduct medical chart review. However, we believe that it still plays an important role in ensuring accountability and improving the quality of health care that we provide.

## CONFLICT OF INTEREST

The authors have declared that no competing interests exist.
